# Impact of Diabetes Duration on Major Adverse Cardiac Events in Patients with Non-Obstructive Coronary Artery Disease

**DOI:** 10.3390/jcm14082797

**Published:** 2025-04-18

**Authors:** Yun-Ah Lee, Sang-Wook Song, Se-Hong Kim, Jin Jung, Won-Young Jang, Donggyu Moon, Sung-Ho Her, Ki-Dong Yoo, Keon-Woong Moon, Su Nam Lee

**Affiliations:** 1 Department of Family Medicine, St. Vincent’s Hospital, College of Medicine, The Catholic University of Korea, Seoul 16247, Republic of Korea; 2 Division of Cardiology, Department of Internal Medicine, St. Vincent’s Hospital, College of Medicine, The Catholic University of Korea, Seoul 16247, Republic of Korea; 3 Catholic Research Institute for Intractable Cardiovascular Disease (CRID), College of Medicine, The Catholic University of Korea, Seoul 06591, Republic of Korea

**Keywords:** non-obstructive coronary artery disease, diabetes mellitus, diabetes duration, long-term clinical outcome

## Abstract

**Background/Objectives:** Diabetes mellitus is a substantial risk factor for coronary artery disease (CAD). Diabetes duration is linked to clinical outcomes in CAD patients. This study aimed to investigate the impact of diabetes duration on major adverse cardiovascular and cerebrovascular outcomes, as well as all-cause mortality, in Korean patients diagnosed with non-obstructive CAD. **Methods:** This non-randomized, retrospective, single-center study was based on the medical records of 4287 patients who underwent coronary angiography from 1 January 2010 to 31 December 2015. Of these patients, 517 with non-obstructive CAD—defined as 20–49% coronary artery stenosis—were identified and categorized into three groups based on diabetes duration: those without diabetes, those with diabetes for <10 years, and those with diabetes for ≥10 years. **Results:** Over a median follow-up period of 60 months, the risk of major adverse cardiovascular and cerebrovascular events (MACCEs) increased nearly fourfold in patients who had non-obstructive CAD and diabetes for ≥10 years compared to those without diabetes, even after adjusting for covariates (adjusted hazard ratio [HR] 4.61, 95% confidence interval [CI] 2.04–10.40, *p* < 0.001). The risks of cardiovascular death and non-fatal stroke were also significantly higher in patients who had diabetes for ≥10 years compared to non-diabetic patients (adjusted HR 12.42, 95% CI 2.33–66.22, *p* = 0.003, adjusted HR 4.97, 95% CI 1.88–13.19, *p* = 0.001, respectively). **Conclusions:** Patients with non-obstructive CAD and a longer duration of diabetes exhibited a higher risk of MACCEs. Diabetes duration could be an important factor in predicting mortality in patients with non-obstructive CAD.

## 1. Introduction

The global prevalence of type 2 diabetes is high and continues to rise across all regions [1]. The International Diabetes Federation estimates that approximately 537 million people are affected by diabetes globally, with type 2 diabetes accounting for over 90% of these cases. These figures are projected to increase to 643 million by 2030 and 783 million by 2045 [2]. Diabetes mellitus substantially heightens the risk of developing coronary artery disease (CAD), exacerbates its complexity, and worsens clinical outcomes [3,4,5]. Moreover, CAD is the leading cause of death and disease burden in individuals with diabetes [6].

Diabetes is a chronic, progressive condition known for causing long-term microvascular and macrovascular complications in various organs. Consequently, diabetes duration has a direct correlation with the clinical outcomes of CAD patients [7]. Previous research has indicated that a prolonged duration of type 2 diabetes is associated with an increased risk of cardiovascular and all-cause mortality among patients undergoing coronary angiography [7,8]. Notably, adverse cardiovascular events and the risk of asymptomatic CAD significantly increase in patients with diabetes for >10.5 years and a systolic blood pressure of >140 mmHg. These factors are predictive of significant coronary stenosis and serve as criteria for recommending cardiovascular screening in individuals with type 2 diabetes [9].

The absence of obstructive CAD is commonly observed in patients undergoing angiography for both acute and chronic coronary syndromes [10,11]. However, the clinical significance of non-obstructive CAD remains a topic of ongoing debate. While initial findings indicated that the absence of obstructive CAD signaled a positive cardiovascular prognosis, subsequent research has suggested that non-obstructive CAD might predict a less favorable outcome compared to the complete absence of CAD [10,12,13,14,15]. This prognostic uncertainty served as a key motivation for exploring the relationship between diabetes duration and the risk of major cardiovascular and cerebrovascular events in patients with non-obstructive CAD.

Limited research has been conducted on the long-term effects of diabetes duration on adverse cardiovascular outcomes in patients with non-obstructive CAD, particularly in Korea. We hypothesized that a longer diabetes duration would be associated with an increased risk of MACCEs in patients with non-obstructive CAD. Therefore, this study sought to assess the impact of diabetes duration on major adverse cardiovascular and cerebrovascular outcomes, as well as all-cause mortality, in Korean adults with non-obstructive CAD.

## 2. Methods

### 2.1. Study Design and Population

**Figure 1:**
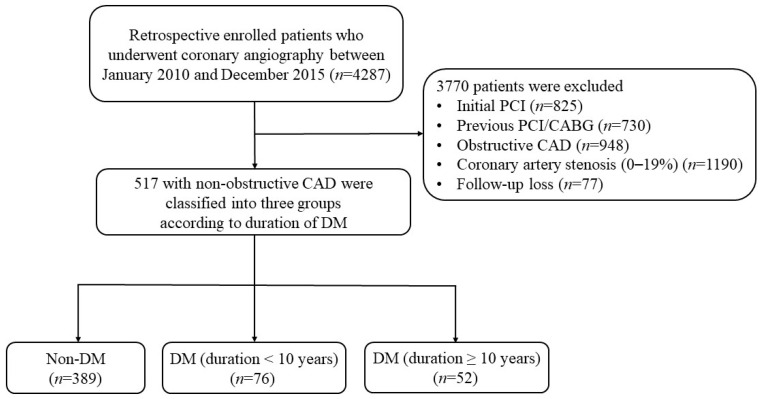
Study population: flow chart of patients who underwent coronary angiography from 1 January 2010 to 31 December 2015, categorized by non-obstructive coronary artery disease (CAD) status, presence of diabetes mellitus (DM), and diabetes duration.

This retrospective, non-randomized study was conducted at a single center using data from 4287 patients who underwent coronary angiography at St. Vincent’s Hospital, Suwon, Republic of Korea, from 1 January 2010 to 31 December 2015. The coronary angiography was performed using Artis zee floor (Siemens Healthineers, Erlangen, Germany) when chest pain occurred or abnormal findings were observed in a transthoracic echocardiogram, an electrocardiogram, a coronary artery calcium scan, a coronary computed tomography angiography (CCTA), or treadmill tests. The extent and severity of CAD were determined by visual assessment performed by the attending interventional cardiologists at the time of the angiography. Non-obstructive CAD was defined as CAD with 20–49% stenosis, while obstructive CAD was defined as ≥50% stenosis, based on the previous published literature and consistent with definitions used in large cohort studies and guideline-based classifications [16,17,18]. The data of 4287 patients who underwent coronary angiography from 1 January 2010 to 31 December 2015 were reviewed. We excluded patients who initially underwent percutaneous coronary intervention (PCI) (*n* = 825), had a history of PCI or coronary artery bypass graft (CABG) surgery (*n* = 730), were diagnosed with obstructive CAD (*n* = 948), had 0–19% stenosis of the coronary artery (*n* = 1190), or were lost to follow-up (*n* = 77) (Figure 1). Consequently, this study focused on the data of 517 participants categorized into three groups based on diabetes duration: those without diabetes, those with diabetes for <10 years, and those with diabetes for ≥10 years (Figure 1). Diabetes duration was determined based on self-reported history, medical records, and medication history documented at the time of coronary angiography. The Institutional Review Board (IRB) of the Catholic University of Korea St. Vincent’s Hospital approved this study and waived the requirement for informed consent (IRB approval number: VC20RISI0087). All procedures were conducted in accordance with the relevant guidelines and regulations. The median follow-up period was 60 months, during which major adverse cardiovascular and cerebrovascular outcomes and all-cause mortality were assessed.

### 2.2. Study Endpoint and Definition

The primary endpoint of this study was the occurrence of major adverse cardiovascular and cerebrovascular events (MACCEs), including cardiovascular death, non-fatal myocardial infarction (MI), and non-fatal stroke. Cardiovascular death was defined as death resulting from MI, sudden cardiac death, heart failure, stroke, or other vascular causes. Secondary endpoints included the individual components of the primary outcome and all-cause mortality. Stroke was identified by the presence of neurological deficits, which was confirmed by imaging findings on either computed tomography or magnetic resonance imaging. Follow-up information, including survival status and clinical outcomes, was obtained up to 27 April 2022 by trained reviewers blinded to the study outcomes, and data collection was conducted through hospital chart reviews and patient telephone interviews. The period from study enrollment to the occurrence of the first event was used to calculate the time-to-event duration. Cigarette smoking status was defined as smoking within the 3 months prior to hospitalization, following definitions used in prior cardiovascular studies [19]. Chronic kidney disease (CKD) was defined as an estimated glomerular filtration rate below 60 mL/min/1.73 m^2^, calculated using the Modification of Diet in Renal Disease equation based on baseline serum creatinine levels [20].

### 2.3. Statistical Analysis

Continuous variables are presented as the mean ± standard deviation and were compared using Student’s *t*-test. Categorical variables are reported as counts (percentages) and were evaluated using the chi-square test or Fisher’s exact test, as appropriate. Event rates were estimated using the Kaplan–Meier method in time-to-first event analysis and were analyzed through the log-rank test. To examine clinical outcomes, univariable and multivariable Cox regression analyses were employed. Hazard ratios (HRs) and 95% confidence intervals (CIs) were calculated. The multivariable Cox regression models were adjusted for several variables. Variables with significant associations in univariate analysis (*p* < 0.05) were selected as covariates, and the stepwise method was used. In addition to crude HRs, adjusted HRs were estimated after the adjustment for covariates. Model 1 was adjusted for age and sex. Model 2 was adjusted for model 1 plus body mass index, hypertension, chronic kidney disease, creatinine, hemoglobin, LDL-cholesterol, and C-reactive protein. The SAS software (version 9.2; SAS Institute, Cary, NC, USA) was used for all statistical analyses, with *p* < 0.05 considered as statistically significant.

## 3. Results

### 3.1. Baseline Characteristics

This study analyzed data from 517 patients with non-obstructive CAD categorized into three groups based on diabetes duration: those without diabetes (*n* = 389, 75.2%), those with diabetes for <10 years (*n* = 76, 14.7%), and those with diabetes for ≥10 years (52, 10.1%). The average age of participants was 63.0 ± 11.2 years, with men comprising 52.2% (*n* = 270) of the population. Baseline characteristics, including age, body mass index, hypertension and CKD history, cilostazol usage, left ventricular ejection fraction, fasting glucose, glycated hemoglobin (HbA1c), creatinine, hemoglobin, hematocrit, total cholesterol, triglycerides, HDL-cholesterol, LDL-cholesterol, C-reactive protein, and albumin levels, varied significantly among the groups. Notably, patients with diabetes for ≥10 years were generally older and were more likely to have CKD, a lower left ventricular ejection fraction, and lower HDL-cholesterol levels, as well as higher levels of C-reactive protein, fasting glucose, and HbA1c (Table 1).

### 3.2. Clinical Outcomes

**Figure 2:**
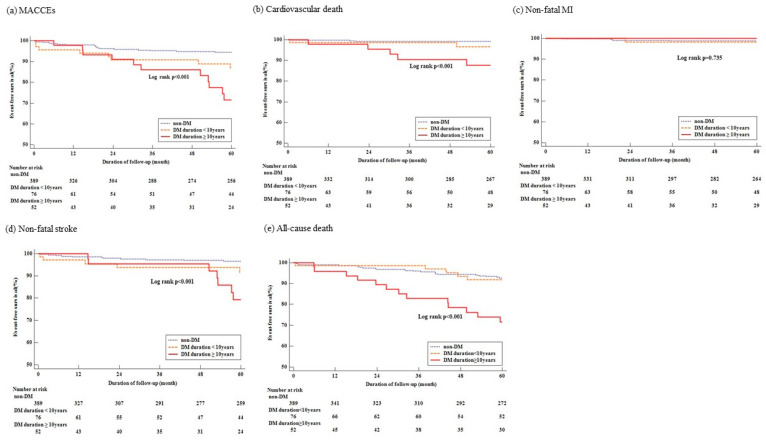
Kaplan–Meier curves showing the event-free survival rates for (**a**) major adverse cardiac and cerebrovascular events (MACCEs), (**b**) cardiovascular death, (**c**) non-fatal myocardial infarction (MI), (**d**) non-fatal stroke, and (**e**) all-cause death.

Over a median follow-up of 60 months, 38 (7.4%) patients experienced MACCEs, with incidence rates differing statistically among groups. Patients with diabetes ≥10 years exhibited the highest rate of MACCEs (Table 2, Figure 2). When compared to the non-diabetic reference group, the risk of MACCEs in patients with diabetes for ≥10 years increased more than fourfold after adjusting for covariates (adjusted HR 4.61, 95% CI 2.04–10.40, *p* < 0.001) (Table 3).

Regarding secondary outcomes, all-cause mortality was observed in 43 (8.3%) patients, cardiovascular death in 10 (1.9%), and non-fatal stroke in 23 (4.4%) (Table 2). Statistically significant differences in the incidence rates of all-cause mortality, cardiovascular death, and non-fatal stroke were noted among the groups. Patients with diabetes for ≥10 years had the highest rates of all-cause mortality, cardiovascular death, and non-fatal stroke (Table 2). Especially, the risks of cardiovascular death and non-fatal stroke were significantly higher in patients with diabetes for ≥10 years compared to those without diabetes, even after adjustments (adjusted HR 12.42, 95% CI 2.33–66.22, *p* = 0.003, adjusted HR 4.97, 95% CI 1.88–13.19, *p* = 0.001, respectively) (Table 3).

## 4. Discussion

This study explored the relationship between diabetes duration and the incidence rates of MACCEs and all-cause mortality among patients with non-obstructive CAD. We found that non-obstructive CAD patients with diabetes for ≥10 years had a significantly higher risk of MACCEs compared to those without diabetes. Specifically, during a median follow-up of 60 months, the risk of MACCEs was almost four times higher in patients with diabetes for ≥10 years, even after adjusting for various covariates. Additionally, the adjusted risk for cardiovascular death and non-fatal stroke was notably higher in patients with diabetes for ≥10 years compared to those without diabetes.

Participants in this study were diagnosed with non-obstructive CAD following initial coronary angiography and had not undergone prior revascularization or PCI. Despite the frequency of non-obstructive CAD cases in clinical settings, their significance might be overlooked as these patients are typically considered low risk for adverse events, and current guidelines for their management are lacking. Non-obstructive CAD, characterized by atherosclerotic plaques not expected to restrict blood flow or cause angina, is common, being found in 10–25% of coronary angiography patients. Traditionally, such findings have been deemed “insignificant” or indicative of “no significant CAD” [17,21] in the medical literature. However, this understanding of non-obstructive CAD may be misleading, as evidence suggests that plaque ruptures leading to MIs often originate from non-obstructive lesions [14,22,23]. Adverse cardiovascular events in patients with non-obstructive CAD may be explained by several underlying pathophysiological mechanisms. As supported by Libby and Theroux, non-obstructive coronary lesions can contribute to major adverse cardiovascular events through mechanisms such as plaque rupture, thrombosis, endothelial dysfunction, and systemic inflammation, highlighting their clinical importance despite the absence of significant stenosis [23]. The fractional flow reserve can be used to evaluate the functional impact of coronary artery lesions, supplementing angiographic data to guide treatment decisions in CAD patients [24]. However, in many clinical settings, decisions are frequently based solely on angiographic findings due to limitations related to equipment availability, insurance coverage, and financial constraints. This study reflects such real-world clinical practices.

Several studies have established a significant connection between diabetes duration and adverse cardiovascular outcomes, highlighting that the risk of chronic vascular complications escalates with both the duration and severity of hyperglycemia [25]. A notable prospective, two-center study involving 933 asymptomatic type 2 diabetic patients who did not have known CAD and underwent CCTA found that a longer diabetes duration was associated with a greater extent and prevalence of CAD. This study also noted a significantly higher risk of MACCEs in these asymptomatic diabetic patients [25]. Similarly, a prospective study focusing on men aged 60–79 years indicated that only an early onset of diabetes lasting >10 years is considered equivalent to CAD [26]. Kim et al. uncovered a significant correlation between diabetes duration and left ventricular diastolic function [27]. The Coronary Artery Risk Development in Young Adults study, which tracked 3628 participants aged 18–30 years without diabetes or prediabetes at baseline, found that the duration of diabetes and prediabetes in adulthood was independently linked to subclinical atherosclerosis and left ventricular systolic and diastolic dysfunction in middle age [28]. Furthermore, the Framingham Heart Study revealed that for each 10-year increase in diabetes duration, the risk of CAD rose by 1.38 times, and the risk of CAD death increased by 1.86 times, regardless of other coexisting risk factors [29]. In alignment with these findings, our study adds novel long-term evidence on the clinical impact of diabetes duration on MACCEs and all-cause mortality in patients who underwent coronary angiography and were identified as having non-obstructive CAD, defined as CAD with 20–49% stenosis. Our results showed that the risk of MACCEs increased more than fourfold in non-obstructive CAD patients with diabetes for ≥10 years compared with those without diabetes. Furthermore, the risk of cardiovascular death and non-fatal stroke significantly increased in patients with ≥10 years of diabetes, highlighting the critical importance of diabetes duration as a factor in cardiovascular risk assessment and management in patients with non-obstructive CAD. Given the significantly elevated risk of adverse cardiovascular and cerebrovascular events observed in patients with non-obstructive CAD and longer diabetes duration, there is a clinical need for more proactive and targeted risk stratification in this population. Particularly, participants with a diabetes duration of ≥10 years had a high incidence of non-fatal stroke, which suggests that more thorough examinations using tests such as carotid ultrasound and electrocardiography (ECG) should be considered to evaluate atrial fibrillation in patients with non-obstructive CAD. Supplementary assessments such as resting or stress ECG and coronary artery calcium scoring could further enhance risk stratification. These tools may help to identify high-risk individuals who might otherwise be underestimated based on angiographic findings alone. Implementing such strategies could support more personalized preventive interventions in diabetic patients, particularly those with ≥10 years of duration.

Diabetes duration has been shown to increase the risk of microalbuminuria, and the risk of CAD is significantly impacted by diabetes duration in patients with nephropathy [30,31]. Individuals with a longer history of diabetes tend to develop atherosclerotic lesions, heightening the risk of CAD death during coronary events [29]. Long-term hyperglycemia may lead to endothelial dysfunction, thereby increasing susceptibility to CAD [32]. Diabetes, which is linked to systemic oxidative stress, may contribute to an elevated risk of CAD and subsequent mortality in patients due to prolonged exposure to oxidative stress as the diabetes duration increases [33]. Additionally, extended hyperglycemia can cause the nonenzymatic glycosylation of proteins in the myocardium and arterial wall [34]. A more extended diabetes duration is associated with autonomic neuropathy and decreased heart rate variability, potentially raising the risk of cardiovascular death. Furthermore, diabetes has been connected to abnormalities in coagulation mechanisms, suggesting an increased risk of acute thrombosis [35,36].

This study marks the first investigation of the relationship between diabetes duration and clinical outcomes in South Korean patients diagnosed with non-obstructive CAD through coronary angiography. However, several limitations should be noted. First, the observational and retrospective design complicates the establishment of causality in the observed associations. Potential unmeasured confounding variables could have introduced bias into the results. While efforts were made to mitigate this by adjusting for known risk factors, not all confounding factors influencing cardiovascular outcomes and mortality could be accounted for. Second, the impact of this study is constricted by its relatively small sample. Third, the low incidence of cardiac events observed over the 5-year follow-up period restricts the depth of interpretation. Fourth, while non-obstructive CAD was defined as 20–49% stenosis, this range may include lesions that clinically depend on their anatomical location, such as the left main artery or the proximal left anterior descending artery. As lesion-specific location data were not available in this study, we were unable to assess the potential differential impact of high-risk lesion locations on outcomes. Fifth, we did not include information related to whether patients had type 1 or 2 diabetes and the diabetes treatment of the participants. Diabetes medications such as sodium–glucose cotransporter 2 inhibitors and glucagon-like-peptide-1 receptor agonists are known to exhibit favorable cardioprotective effects and could potentially impact the observed associations. Although baseline HbA1c levels were assessed, data on long-term glycemic control during the follow-up period were not available, which may have influenced cardiovascular outcomes. Lastly, detailed information on socioeconomic status was lacking, and this factor may serve as a potential confounder that could not be fully adjusted for in the analysis. In addition, data on key lifestyle factors such as dietary habits, physical activity, smoking cessation efforts, and adherence to statins were not available. These unmeasured variables may have influenced cardiovascular outcomes and represent important potential confounders that warrant consideration in future studies. Consequently, further research with a larger cohort and extended follow-up is necessary to enhance the robustness and clarity of these findings.

## 5. Conclusions

The findings of this study suggest that a longer diabetes duration in non-obstructive CAD patients is linked to a significantly increased risk of MACCEs, cardiovascular death, and non-fatal stroke. These results underscore the clinical significance of managing non-obstructive CAD in patients with an extended history of diabetes and advocate for further investigation into interventions aimed at improving outcomes for this population. Physicians should note that diabetes duration might be an important predictor of mortality in patients with non-obstructive CAD.

## Figures and Tables

**Table 1:** The baseline characteristics of participants according to the duration of diabetes mellitus.

	Non-DM	DM_Duration < 10 Years	DM_Duration ≥ 10 Years	*p*-Value
*N*	389	76	52	-
DM duration (years)		3 (2–6)	15 (10–20)	
Age (years)	62.2 ± 11.5	64.0 ± 9.9	68.1 ± 9.8	0.001
Male	208 (53.5)	34 (44.7)	28 (53.9)	0.557
BMI (kg/m^2^)	24.3 ± 3.0	25.7 ± 3.6	24.0 ± 3.2	0.002
Hypertension	192 (49.4)	53 (69.7)	35 (67.3)	<0.001
Dyslipidemia	78 (20.1)	20 (26.3)	8 (15.4)	0.292
Smoking	133 (34.2)	20 (26.3)	13 (25.0)	0.207
CKD	2 (0.5)	3 (4.0)	5 (9.6)	<0.001
Previous CVA	26 (6.7)	6 (7.9)	3 (5.8)	0.887
Aspirin	213 (54.8)	39 (51.3)	22 (42.3)	0.228
Thienophyridine	26 (6.7)	7 (9.2)	3 (5.8)	0.686
Cilostazol	10 (2.6)	2 (2.6)	8 (15.4)	<0.001
Statin	138 (35.5)	26 (34.2)	15 (28.9)	0.639
Beta blocker	62 (15.9)	15 (19.7)	11 (21.2)	0.510
ACEi/ARB	69 (17.7)	20 (26.3)	14 (26.9)	0.095
Vasodilator	157 (40.4)	27 (35.5)	17 (32.7)	0.459
LV EF (%)	61.5 ± 8.5	60.7 ± 9.8	56.7 ± 11.4	0.008
Fasting glucose (mg/dL)	113.4 ± 34.2	153.5 ± 51.5	183.7 ± 96.6	<0.001
HbA1c (%)	5.9 ± 0.6	7.3 ± 1.2	7.9 ± 1.7	<0.001
Creatinine (mg/dL)	0.8 (0.7–1.0)	0.8 (0.7–1.0)	0.9 (0.7–1.3)	0.018
Hemoglobin (g/dL)	13.7 ± 1.8	13.6 ± 1.9	12.8 ± 1.8	0.005
Hematocrit (%)	40.1 ± 4.5	39.6 ± 5.2	37.3 ± 5.0	<0.001
Platelet (×10^9^/L)	237.6 ± 60.7	254.4 ± 72.6	231.8 ± 64.3	0.078
White blood cell (×10^9^/L)	7.7 ± 2.8	7.7 ± 3.1	7.5 ± 2.3	0.886
Total cholesterol (mg/dL)	188.4 ± 37.4	174.7 ± 47.7	160.9 ± 40.3	<0.001
Triglycerides (mg/dL)	125.1 ± 75.8	157.0 ± 92.3	129.4 ± 94.5	0.013
HDL-cholesterol (mg/dL)	44.2 ± 12.1	41.2 ± 10.3	37.6 ± 11.2	0.002
LDL-cholesterol (mg/dL)	114.6 ± 32.9	99.6 ± 40.9	96.0 ± 32.0	<0.001
C-Reactive protein (mg/dL)	0.13 (0.06–0.34)	0.18 (0.08–0.42)	0.28 (0.09–1.53)	0.031
Albumin (g/dL)	4.3 ± 0.4	4.2 ± 0.5	4.1 ± 0.4	0.007

Values are means ± standard deviation or number (%); DM, diabetic mellitus; BMI, body mass index; CKD, chronic kidney disease; CVA, cerebrovascular accident; LV EF, left ventricular ejection fraction; ACEi, angiotensin-converting–enzyme inhibitor; ARB, angiotensin II receptor blocker; HbA1c, glycated hemoglobin; HDL, high-density lipoprotein; LDL, low-density lipoprotein.

**Table 2:** The 5-year clinical outcomes of participants according to the duration of diabetes mellitus.

	Non-DM (*n* = 389)	DM_Duration < 10 Years (*n* = 76)	DM_Duration ≥ 10 Years (*n* = 52)	*p*-Value
MACCEs	18 (4.6)	8 (10.5)	12 (23.1)	<0.001
All-cause death	25 (6.4)	5 (6.6)	13 (25.0)	<0.001
Cardiovascular death	3 (0.8)	2 (2.6)	5 (9.6)	<0.001
Non-fatal MI	4 (1.0)	1 (1.3)	0 (0.0)	0.734
Non-fatal stroke	11 (2.8)	5 (6.6)	7 (13.5)	<0.001

Values are shown as *n* (%) DM, diabetic mellitus; MACCEs, major adverse cardiovascular and cerebrovascular events; MI, myocardial infarction.

**Table 3:** The 5-year hazard ratios of MACCEs, all-cause death, cardiovascular death, non-fatal myocardial infarction, and non-fatal stroke.

	Unadjusted HR (95% CI)	*p*-Value	Model 1	*p*-Value	Model 2	*p*-Value
MACCEs						
non-DM	1.00	-	1.00	-	1.00	
DM_duration < 10 years	2.45 (1.07–5.64)	0.035	2.48 (1.08–5.71)	0.033	2.34 (0.98–5.55)	0.055
DM_duration ≥ 10 years	5.39 (2.59–11.20)	<0.001	5.52 (2.58–11.81)	<0.001	4.61 (2.04–10.40)	<0.001
All-cause death						
non-DM	1.00	-	1.00	-	1.00	
DM_duration < 10 years	1.05 (0.40–2.74)	0.923	0.97 (0.37–2.53)	0.946	0.88 (0.33–2.37)	0.796
DM_duration ≥ 10 years	4.16 (2.13–8.14)	<0.001	3.38 (1.70–6.74)	<0.001	2.09 (0.94–4.64)	0.069
Cardiovascular death						
non-DM	1.00	-	1.00	-	1.00	
DM_duration < 10 years	3.57 (0.60–21.34)	0.164	3.60 (0.60–21.58)	0.162	3.01 (0.41–22.16)	0.281
DM_duration ≥ 10 years	13.19 (3.15–55.24)	<0.001	13.57 (3.07–60.04)	<0.001	12.42 (2.33–66.22)	0.003
Non-fatal MI						
non-DM	1.00	-	1.00	-	1.00	
DM_duration < 10 years	1.31 (0.15–11.74)	0.808	1.54 (0.17–14.12)	0.703	1.14 (0.11–12.04)	0.911
DM_duration ≥ 10 years	-	-	-	-	-	
Non-fatal stroke						
non-DM	1.00	-	1.00	-	1.00	
DM_duration < 10 years	2.54 (0.88–7.31)	0.084	2.49 (0.86–7.18)	0.091	2.21 (0.74–6.62)	0.158
DM_duration ≥ 10 years	6.10 (2.45–15.18)	<0.001	5.53 (2.15–14.23)	<0.001	4.97 (1.88–13.19)	0.001

Model 1: Hazard ratios adjusted by age and sex. Model 2: Hazard ratios adjusted by model 1 plus body mass index, hypertension, chronic kidney disease, creatinine, hemoglobin, LDL-cholesterol, and C-reactive protein. HR, hazard ratio; CI, confidence interval; DM, diabetic mellitus; MACCEs, major adverse cardiovascular and cerebrovascular events; MI, myocardial infarction.

## Data Availability

The data that support the results of this study are available from the corresponding author upon reasonable request.
